# Behavior of Gingival Fibroblasts on Titanium Implant Surfaces in Combination with either Injectable-PRF or PRP

**DOI:** 10.3390/ijms18020331

**Published:** 2017-02-04

**Authors:** Xuzhu Wang, Yufeng Zhang, Joseph Choukroun, Shahram Ghanaati, Richard J. Miron

**Affiliations:** 1 The State Key Laboratory Breeding Base of Basic Science of Stomatology (Hubei-MOST) & Key Laboratory of Oral Biomedicine Ministry of Education, School & Hospital of Stomatology, Wuhan University, Wuhan 430079, China; wangxuzhu@whu.edu.cn; 2 Department of Oral Implantology, School and Hospital of Stomatology, Wuhan University, Wuhan 430079, China; 3 Pain Clinic, 06000 Nice, France; joseph@a-prf.com; 4 FORM, Frankfurt Oral Regenerative Medicine, Clinic for Maxillofacial and Plastic Surgery, Johann Wolfgang Goethe University, 60596 Frankfurt am Main, Germany; s.ghanaati@med.uni-frankfurt.de; 5 Department of Periodontology, College of Dental Medicine, Nova Southeastern University, Fort Lauderdale, FL 33328, USA; 6 Cell Therapy Institute, Collaborative Centre for Research, Nova Southeastern University, Fort Lauderdale, FL 33328, USA; 7 Department of Periodontics and Oral Surgery, University of Ann Arbor, Ann Arbor, MI 48109, USA

**Keywords:** fibrin, blood, platelets, regeneration, wound healing, fibroblasts, platelet-rich fibrin

## Abstract

Various strategies have been employed to speed tissue regeneration using bioactive molecules. Interestingly, platelet concentrates derived from a patient’s own blood have been utilized as a regenerative strategy in recent years. In the present study, a novel liquid platelet formulation prepared without the use of anti-coagulants (injectable-platelet-rich fibrin, i-PRF) was compared to standard platelet-rich plasma (PRP) with gingival fibroblasts cultured on smooth and roughened titanium implant surfaces. Standard PRP and i-PRF (centrifuged at 700 rpm (60× *g*) for 3 min) were compared by assays for fibroblast biocompatibility, migration, adhesion, proliferation, as well as expression of platelet-derived growth factor (PDGF), transforming growth factor-β (TGF-β), collagen1 (COL1) and fibronectin (FN). The results demonstrate that i-PRF induced significantly higher cell migration, as well as higher messenger RNA (mRNA) levels of PDGF, TGF-β, collagen1 and fibronectin when compared to PRP. Furthermore, collagen1 synthesis was highest in the i-PRF group. These findings demonstrate that liquid platelet concentrates can be formulated without the use of anticoagulants and present much translational potential for future research. Future animal and clinical trials are now necessary to further investigate the potential of utilizing i-PRF for soft tissue regenerative protocols in combination with various biomaterials.

## 1. Introduction

Surface topography of dental implants has undergone numerous modifications over the years to improve their ability to osseointegrate into host tissues [1,2,3,4]. While it has been shown that a roughened surface improves osteoblast differentiation and bone-to-implant contact, additional attempts have more recently been made to coat surfaces with bioactive molecules such as bone morphogenetic proteins, to further speed the quality of new bone formation [5,6,7]. While standard implants do not take advantage of such strategies mainly due to their high associated costs, it remains interesting to note that platelet concentrates derived from the patient’s own blood (autologous) can be harvested at relatively no cost and have been shown to additionally lead to improvements in wound healing [8,9].

While over the past two decades most research has focused on hard tissue integration of dental implants, more recently, soft tissue healing around implants has been the focus of much discussion due to its prominent role in their long-term maintenance [10,11]. Interestingly, it has been demonstrated that platelet concentrates have specifically a more pronounced effect on soft tissue wound healing when compared to hard tissues due to their incorporation of various growth factors including platelet-derived growth factor (PDGF), vascular endothelial growth factor (VEGF) and transforming growth factor-β1 (TGF-β1) [12,13,14]. The first generation of platelet concentrates was pioneered in the dental field by Marx et al. [15,16] utilizing platelet-rich plasma (PRP). Since then, their use has been widespread across many fields of dentistry, orthopaedics and esthetics for the regeneration of tissues based on their ability to increase angiogenesis [15,16,17,18,19]. Despite this, limitations in their regenerative potential have been reported mainly due to their incorporation of anti-coagulants, known suppressors of tissue regeneration [15,20,21].

For these reasons, in 2001 a new formulation of platelet concentrate termed platelet-rich fibrin (PRF) was developed in an attempt to remove anti-coagulants [22]. Since PRF does not contain anti-coagulants, it forms a fibrin clot within minutes after blood collection that has since been described and utilized as a three-dimensional scaffold for tissue regeneration [23,24,25]. Despite having complete immune-biocompatibility, other advantages include faster angiogenesis of tissues, which leads to faster wound healing [26,27,28,29]. For these reasons, the use of PRF has been widespread in oral surgery and continues to grow exponentially in use [30]. One of the main reported limitations of PRF has been its more difficult combination with bone biomaterials, due to its fibrin scaffold consistency, as opposed to a liquid/gel formulation such as that found in PRP.

Recently, an injectable formulation of PRF (i-PRF) has been developed by our group to fulfil this criterion without having to use anti-coagulants; i-PRF is formulated by centrifugation at lower speeds (700 rpm, 60× *g*) for only 3 min and thus must be utilized within 15 min prior to fibrin clot formation. While PRP has previously been utilized in combination with dental implants as a protein coating for implant surfaces [8,9], the aim of this study was to perform a first in vitro study on i-PRF in combination with dental implant surfaces. Therefore, the present study compared i-PRF to the clinically utilized PRP and characterized the behavior of human gingival fibroblast cell viability, migration, proliferation and messenger RNA (mRNA) levels of growth factors (PDGF, TGF-β1, fibronectin and collagen1), as well as collagen1 matrix synthesis.

## 2. Results

### 2.1. Cell Viability

**Figure 1:**
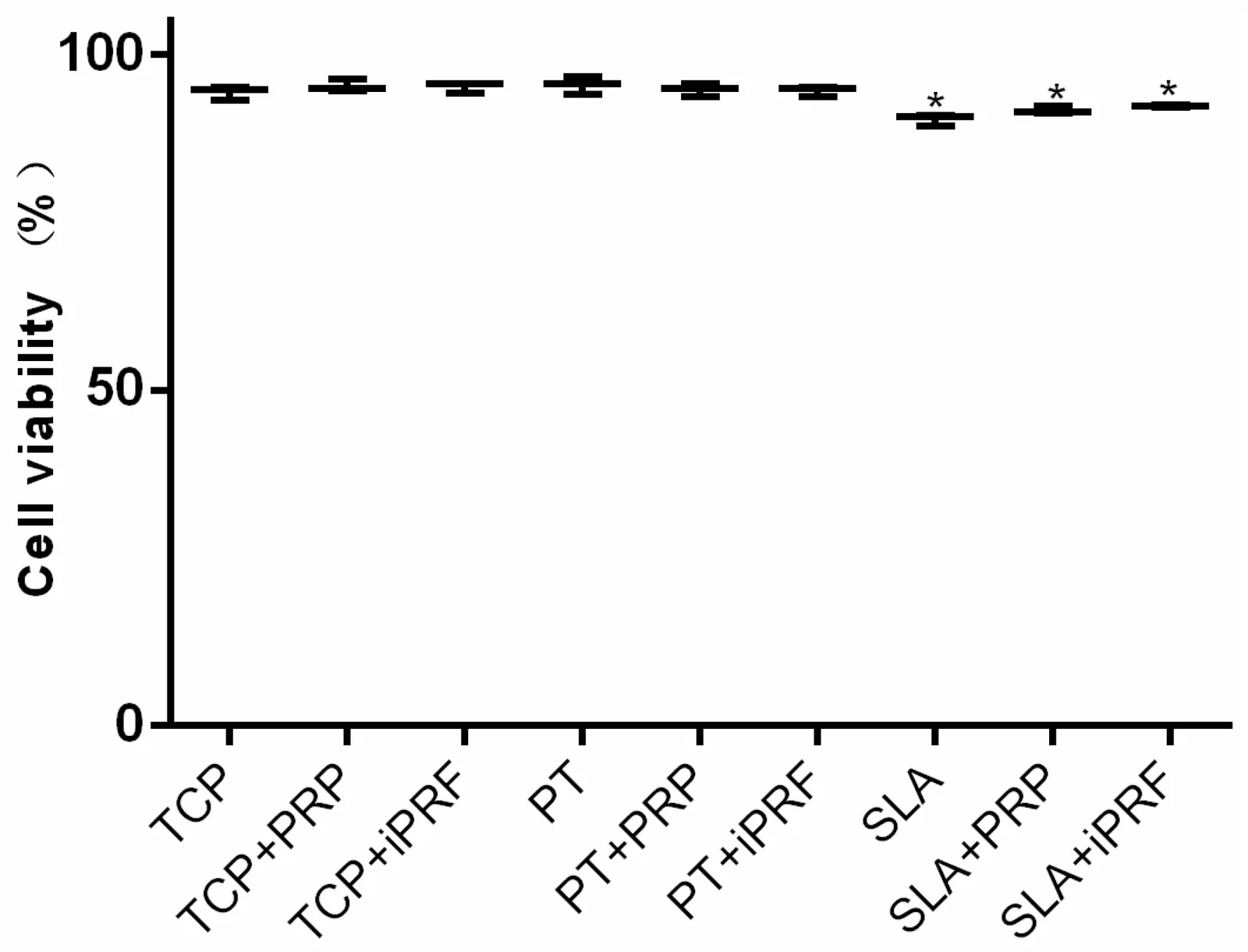
The percentage of human gingival fibroblasts quantified with a Live/Dead assay at 24 h on tissue culture plastic (TCP), Pickled titanium (PT) and Sand-blasted with Large grit particles followed by Acid-etching (SLA) with platelet rich plasma (PRP) or injectable platelet-rich fibrin (i-PRF) (* denotes difference between control TCP and experimental group, *p* < 0.05).

As depicted in Figure 1, the effect of PRP and i-PRF was investigated on cell viability of human gingival fibroblasts. It was found that more than 90% cell survival was observed on all surfaces irrespective of platelet preparations. A slight but significantly lower number of living cells was observed on Sand-blasted with Large grit particles followed by Acid-etching (SLA) surfaces when compared to Pickled titanium (PT) (Figure 1). It may therefore be concluded that both PRP and i-PRF demonstrated excellent cell viability and biocompatibility under the present in vitro culturing conditions.

### 2.2. Cell Migration

**Figure 2:**
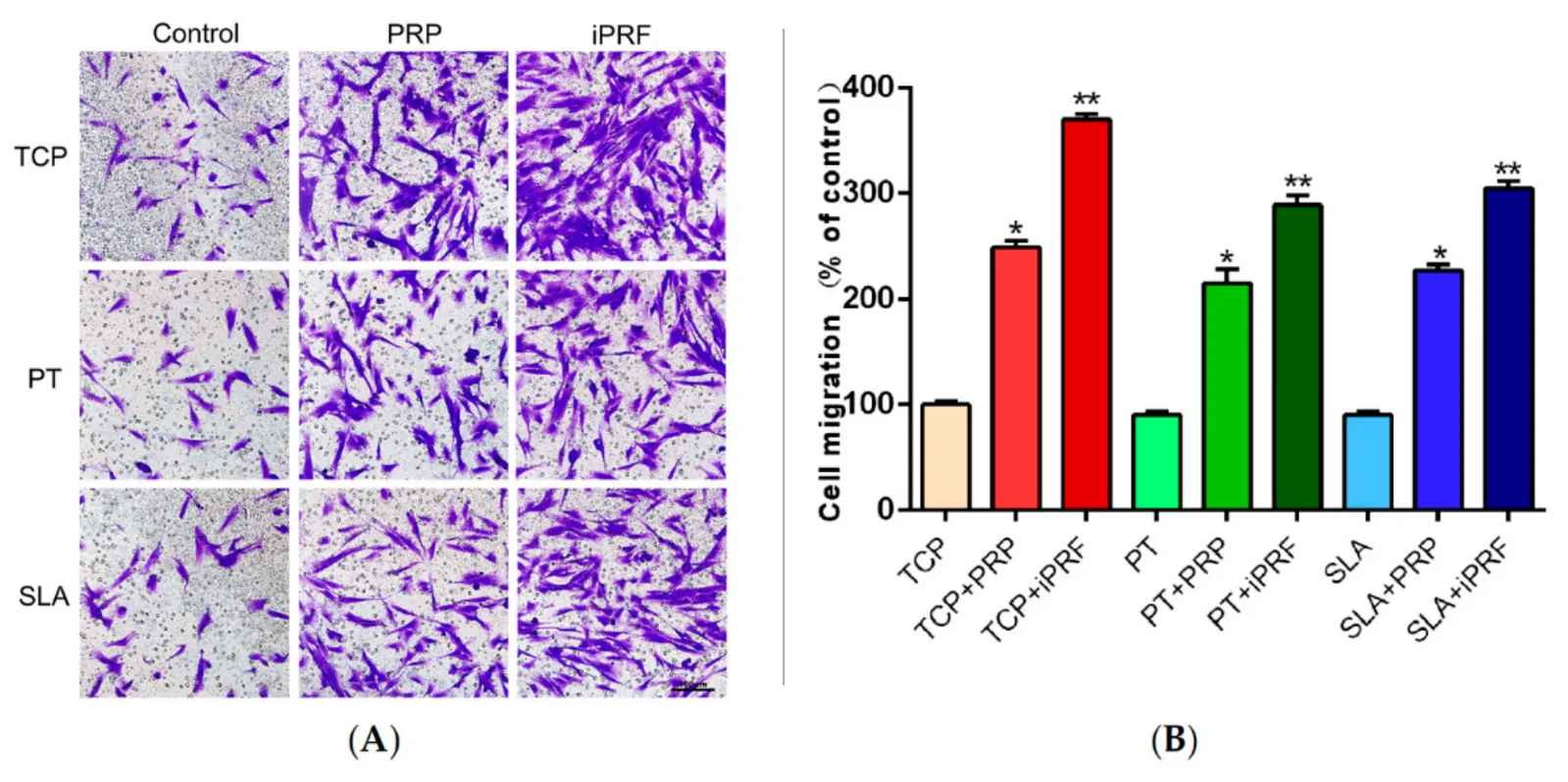
Effects of surface topography and PRP/i-PRF on the migration of human gingival fibroblasts. (**A**) Cell migration was assessed after 24 h. (Scale bars = 100 µm); (**B**) Cell migration was quantified by normalizing to the control TCP group. (* denotes significant difference between control and experimental group, *p* < 0.05; ** denotes significantly higher than all other groups, *p* < 0.05).

Thereafter, it was observed that PRP and i-PRF both promoted the migration of human gingival fibroblasts at 24 h (Figure 2). It was found that PRP induced a 250% significant increase relative to controls, whereas i-PRF induced a more than 350% significant increase in migrated cells when compared to control tissue culture plastic (Figure 2). Similar trends were obtained on both PT and SLA surfaces (Figure 2).

### 2.3. Cell Adhesion, Proliferation and Morphology

**Figure 3:**
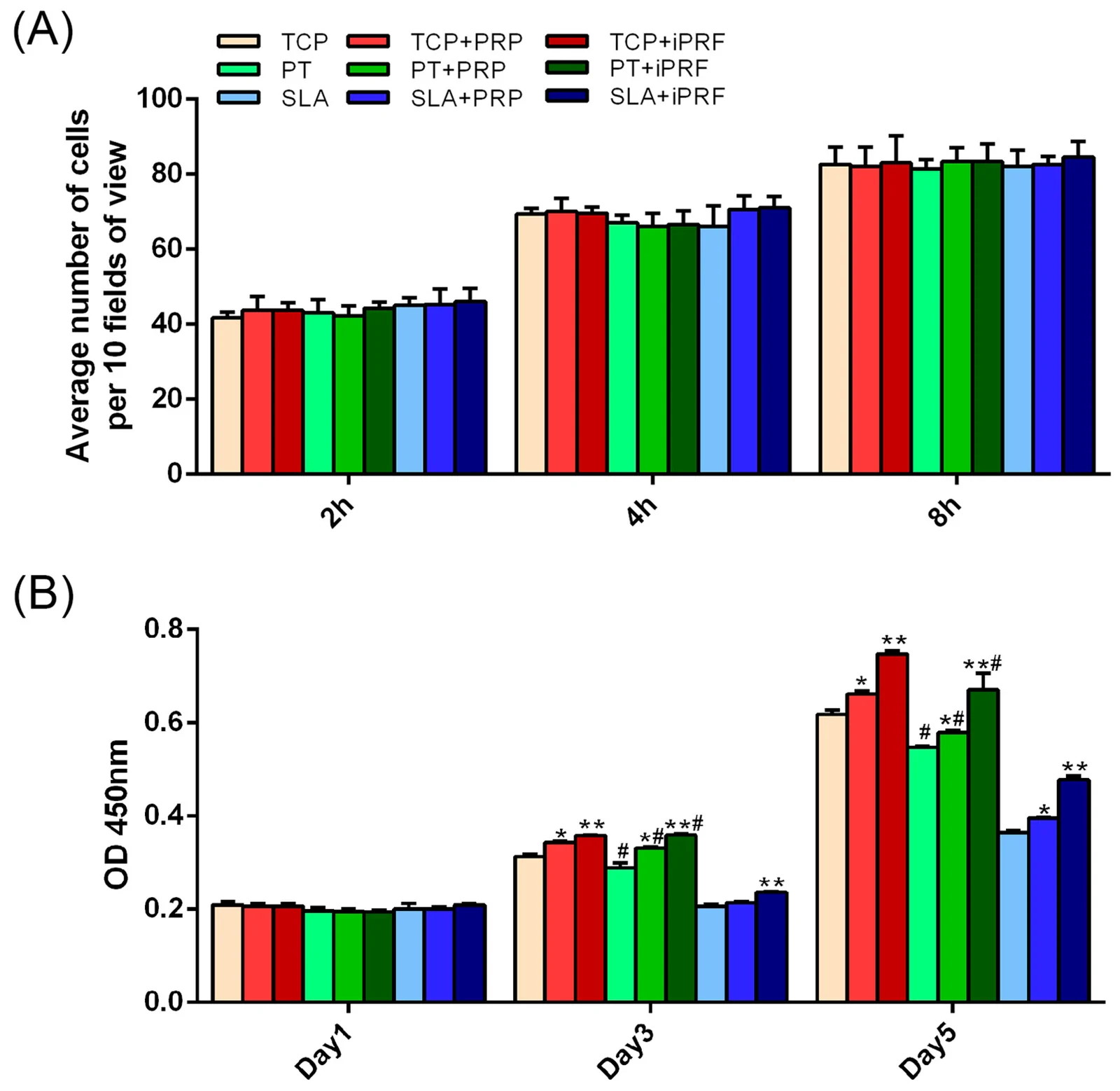
Effect of surface topography in combination with PRP and i-PRF on the adhesion and proliferation of human gingival fibroblasts. (**A**) Cell adhesion at 2, 4 and 8 h and (**B**) Cell proliferation at 1, 3 and 5 days. (* denotes significant difference between control and experimental group, *p* < 0.05; ** denotes significantly higher than all other groups, *p* < 0.05; # denotes significant difference between PT and SLA surfaces *p* < 0.05).

**Figure 4:**
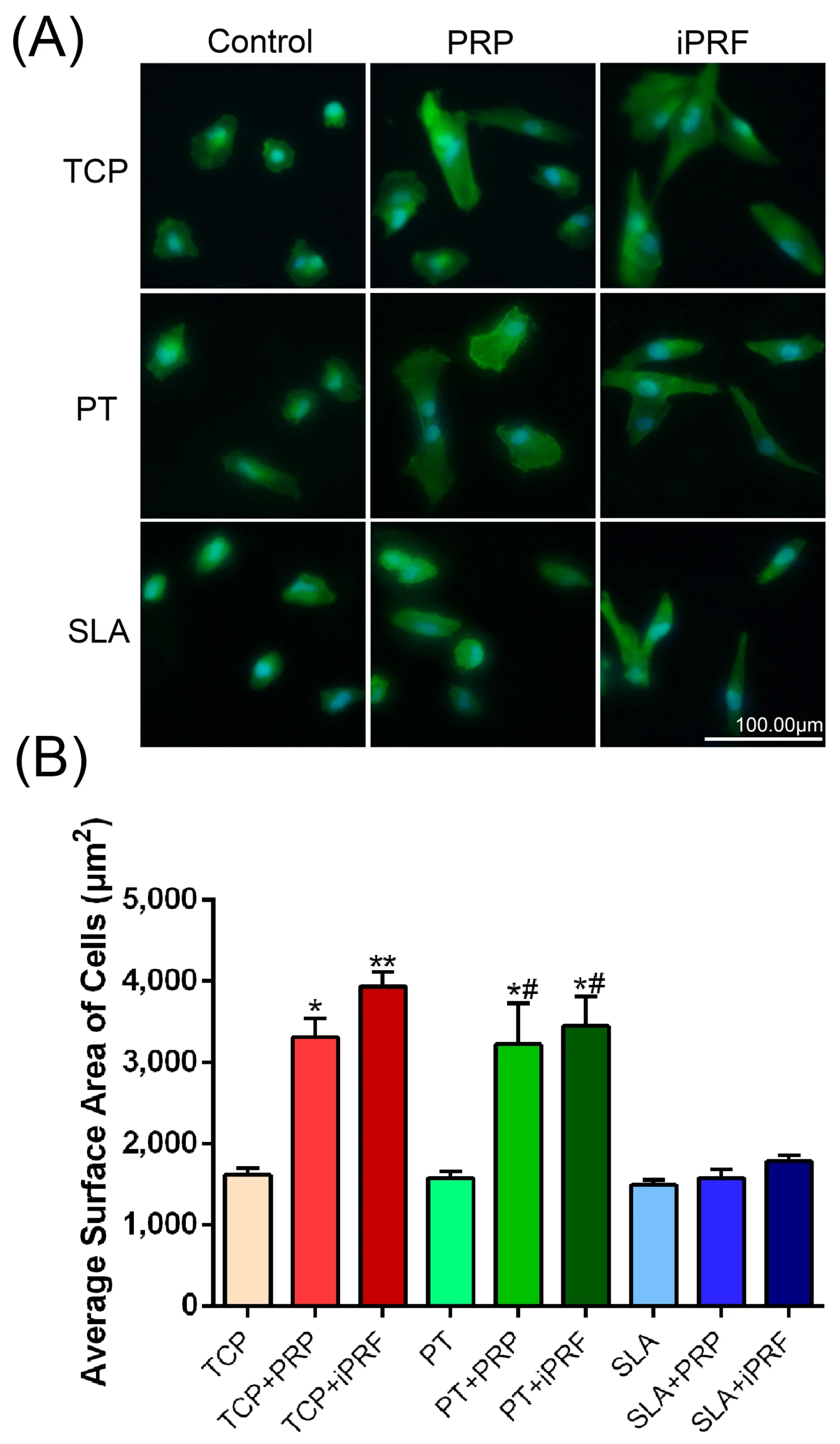
Effects of surface topography in combination with PRP and i-PRF on the morphology of human gingival fibroblasts (**A**) Human gingival fibroblasts cultured with and without PRP or i-PRF at 8 h were stained for F-actin (green) and nuclei (blue) (Scale bars = 100 µm); (**B**) Average surface planar area of cells. (* denotes significant difference between control and experimental group, *p* < 0.05; ** denotes significantly higher than all other groups, *p* < 0.05; # denotes significant difference between PT and SLA surfaces, *p* < 0.05).

For the cell adhesion assays, no significant difference was observed between all surfaces irrespective of surface topography or addition of PRP/i-PRF at all time points including 2, 4 and 8 h (Figure 3A). The morphology of human gingival fibroblasts (HGFs) was then investigated with/without PRP and i-PRF on all surfaces at 8 h. It was found that both PRP and i-PRF promoted HGF cell spreading on tissue culture plastic (TCP) and PT when compared to their respective controls, while cells seeded on SLA surfaces demonstrated less spreading in all groups (Figure 4). Analysis of cell surface area also demonstrated that both PRP and i-PRF increased the cell surface area on TCP and PT but not SLA (Figure 4). Cell proliferation was then investigated (Figure 3B). It was demonstrated that at 1 day post seeding, no significant difference was observed between all groups, regardless of surface topography or the presence of PRP and i-PRF. However, at three and five days post-seeding, it was found that cells cultured specifically on SLA surfaces demonstrated significantly lower cell numbers in comparison to control TCP and control PT surfaces. Meanwhile both PRP and i-PRF significantly increased cell numbers when compared to their respective controls, with i-PRF being significantly higher than all other groups (Figure 3B).

### 2.4. Human Gingival Fibroblast Expression of Regeneration-Related and Extracellular Matrix (ECM)-Related Genes

**Figure 5:**
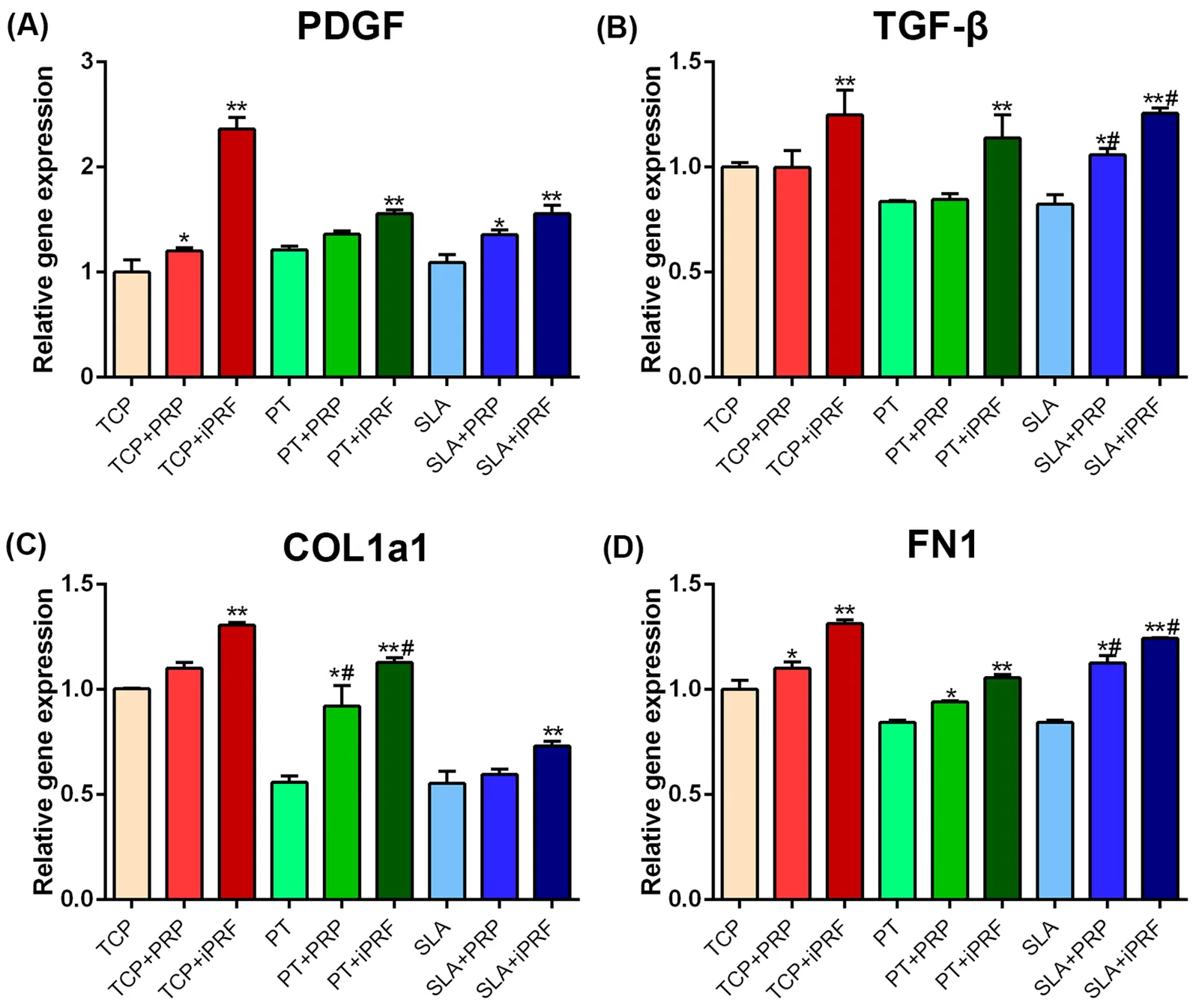
Real-time PCR of human gingival fibroblasts cultured on TCP, PT and SLA, with and without PRP or i-PRF for mRNA levels of (**A**) *PDGF*; (**B**) *TGF-β*; (**C**) *COL1* and (**D**) *FN1*. (* denotes significant difference between control and experimental group, *p* < 0.05; ** denotes significantly higher than all other groups, *p* < 0.05; # denotes significant difference between PT and SLA surfaces, *p* < 0.05).

Next, mRNA levels of regeneration-related genes *PDGF* and *TGF-β* were evaluated by real-time Polymerase Chain Reaction (PCR) (Figure 5A,B). It was found that i-PRF demonstrated significantly highest *PDGF* and *TGF-β* mRNA levels on all surfaces with no differences observed between groups. Thereafter, ECM-related genes including collagen type I α1 (*COL1*) and fibronectin (*FN1*) expression of human gingival fibroblast was also evaluated by real-time PCR (Figure 5C,D). It was found that both PRP and i-PRF provoked an increase in *COL1* and *FN1* mRNA levels when compared to their respective controls, with i-PRF demonstrating significantly highest results (Figure 5C,D).

### 2.5. Collagen Type I Staining

**Figure 6:**
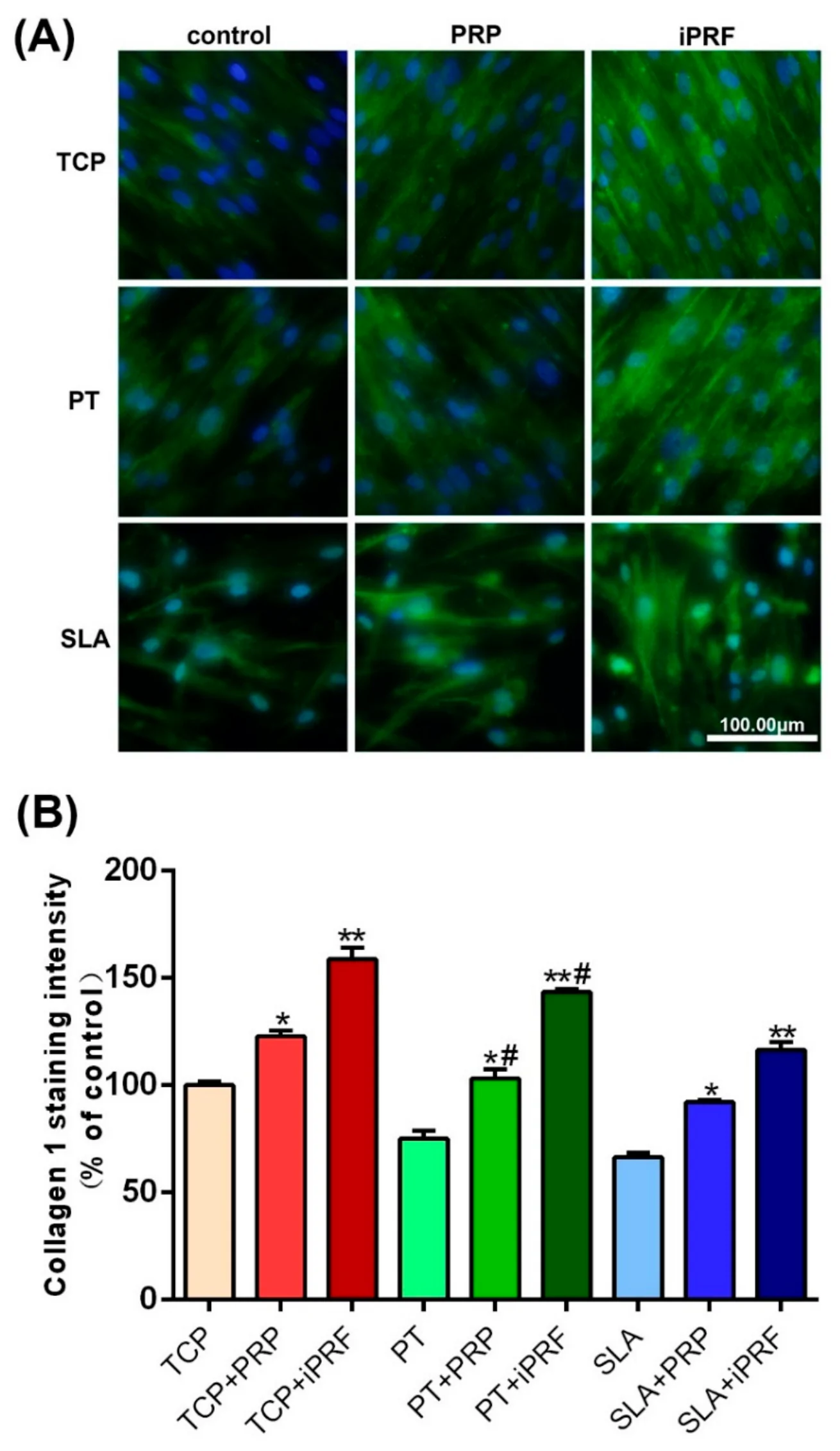
Immunofluorescent staining of collagen type 1 for human gingival fibroblasts seeded on TCP, PT and SLA surfaces with and without PRP and i-PRF at 7 days. (**A**) The merged fluorescent images of collagen type 1 staining (green) with DAPI staining (blue). (Scale bars = 100 µm); (**B**) Quantified values of collagen type 1 staining in comparison to control TCP sample (* denotes significant difference between control and experimental group, *p* < 0.05, ** denotes significantly higher than all other groups, *p* < 0.05, # denotes significant difference between PT and SLA surfaces, *p* < 0.05).

Collagen type 1 immunofluorescent staining was then utilized to confirm the effect of PRP and i-PRF on extra-cellular matrix deposition of human gingival fibroblasts onto titanium surfaces. As presented in Figure 6, it was observed that PRP exhibited a slight increase in immunofluorescent staining of collagen type 1 when compared to their respective controls, while i-PRF demonstrated the greatest fluorescence intensity in collagen type 1 expression on all surfaces. Quantification of staining area confirmed these findings (Figure 6B). Significantly higher total staining was observed on PT surfaces when compared to SLA (Figure 6B).

## 3. Discussion

The aim of the present study was first to investigate and compare the influence of a new platelet concentrate without the use of anti-coagulants (i-PRF in liquid formulation) versus standard PRP on titanium surfaces and, secondly, to investigate if surface topography had an influence on the outcomes. To the best of the authors’ knowledge, this is the first study investigating the influence of i-PRF and, for these reasons, an in vitro study was performed specifically to determine its influence on soft tissue cells (human gingival fibroblasts). As such, an array of in vitro experiments was performed to investigate i-PRF as a potential low-cost regenerative modality derived from 100% autologous sources.

The use of regenerative modalities in dentistry has become increasingly popular in recent years with the use of PRF having seen a plethora of research articles published within the past five years [14,29,31]. As such, many clinicians working in the field of implant dentistry have more recently been made aware of the advantages of utilizing PRF as membranes during guided bone regenerative procedures. Currently, a variety of biomaterials are routinely being commercialized from various xenograft sources including porcine and bovine materials. The use of PRF acts as an entirely autologous biomaterial and thus does not elicit a foreign body reaction and the role of extracellular matrix proteins has been shown to positively contribute to the regeneration process [32].

Interestingly, PRP or preparation rich in growth factor (PRGF) has previously been utilized in the field of implant dentistry as a blood concentrate capable of facilitating various aspects of tissue regeneration when utilized in combination with bone biomaterials in implant dentistry [33]. Anitua et al. [34] (the original pioneers of PRGF) have recently proposed removing anti-coagulants from their platelet formulations after over a decade of use and research including anti-coagulants within its formulation [35]. Remarkably, it has been over 15 years since Choukroun et al. [22] pioneered the field of platelet concentrates without using anti-coagulants and, for these reasons, much further advancements have been made since. Most recently, major development was placed into formulating a liquid version of PRF, termed injectable-PRF, or i-PRF, with the aim of improving biomaterial mixing with platelet concentrates yet additionally forming a fibrin network shortly after bone biomaterial mixing and/or coating favoring biomaterial stability during regenerative procedures.

While both platelet formulations were extremely biocompatible under the present in vitro setting by displaying high levels of cell viability, it was found that i-PRF significantly increased human gingival fibroblast cell migration, proliferation, and spreading. Furthermore, i-PRF promoted the release of pro-wound healing growth factors PDGF and TGF-β, as well as collagen synthesis. Therefore, the lack of anti-coagulants and the natural formulation of i-PRF was shown to possess more optimal regenerative potential on cells under the present in vitro setting.

These findings stem from previous reports that have shown that centrifugation using the “low-speed centrifugation concept” induces less cells to migrate towards the bottom of centrifuge tubes as a result of lower G-forces [13,36]. By decreasing G-force, a higher percentage of cells including platelets and leukocytes remain in the upper compartment of centrifugation tubes where i-PRF is collected, thereby providing more cells capable of assisting in tissue regeneration and the release of pro-wound healing molecules. Previously, our group demonstrated that by lowering centrifugation speed and time, a resulting increase in growth factor release and cell activity was observed [13,33].

These advancements were further improved with i-PRF, whereby even lower centrifugation speeds and times are utilized. These possibilities exist due to the recent findings from Choukroun and Ghanaati, which demonstrated that i-PRF contains a higher proportion of cells including leukocytes prior to the formation of a fibrin clot when compared to other platelet concentrates due to the low centrifugation speeds (Choukroun and Ghanaati, unpublished data, and [36]). While leukocytes are not found in every formulation of platelet concentrates, they are immune cells having shown substantial importance during the host-defence response to incoming pathogens, as well as assisting in the wound healing process by secreting various growth factors [37,38,39].

While the present study remains preliminary and future animal and human research is necessary to fully describe the regenerative potential of i-PRF in medicine and dentistry, we show for the first time that i-PRF significantly increases cell bioactivity when compared to PRP. Therefore, the combination of i-PRF during dental implant placement is thought to provoke faster wound healing, especially in connective tissues. Soft tissue breakdown is thought to be one of the main reasons associated with peri-implantitis and the ability for i-PRF to improve collagen synthesis during the regenerative phase would theoretically improve the ability for host tissues to resist incoming bacterial pathogens. Furthermore, since i-PRF contains leukocytes, its use for the treatment and/or management of peri-implant disease is hypothesized to be of value since leukocytes are thought to actively resist/destroy pathogens found in peri-implantitis. Nevertheless, future clinical research is certainly necessary to validate these hypotheses as the resolution of peri-implant disease remains one of the most challenging faced in modern dentistry.

What also remains to be further investigated is the influence of fibrin formation during i-PRF therapy. It remains unknown what effect this may have during tissue regeneration, although numerous reports have shown that fibrin in general has a positive role on tissue regeneration and new bone formation. For this reason, our group hypothesized that the incorporation of fibrin contained within i-PRF is potentially responsible for more favorably contributing to tissue regeneration when compared to PRP (lacks fibrin) as found in the present study. Furthermore, the larger presence of leukocytes in i-PRF is hypothesized to also further contribute to tissue regeneration by secreting a larger number of growth factors and cytokines necessary for wound healing.

In the present study, it was found that i-PRF was shown to surface topography, seemed to affect the response of human gingival fibroblasts and it may therefore be hypothesized that surface roughness may play a role in i-PRF-induced wound healing. While this is only a hypothesis, the ability for various proteins to adsorb differently to various implant surfaces has been previously investigated by our group, among others [40,41,42,43]. Therefore, the mechanical influence and extracellular matrix proteins adsorbed to the implant surface may play a prominent role in affecting cell behavior [44,45]. Therefore, future research is certainly required to investigate the potential impact on the above-mentioned results of tissue integration. There also remains great interest to further evaluate in vivo in standardized defect models the regenerative potential of i-PRF. While much further research is necessary, this study remains a first of its kind characterizing the influence of i-PRF on human gingival fibroblast behavior in vitro.

## 4. Materials and Methods

### 4.1. PT and SLA Titanium Discs

Commercially pure grade 4 titanium was used to fabricate (1) Pickled titanium (PT) and (2) Sand-blasted with Large grit particles followed by Acid-etching (SLA) surfaces. Titanium discs 15 mm in diameter were provided by Straumann AG (Basel, Switzerland), which fit directly into the bottom of 24-well culture plates. Briefly, smooth PT surfaces were prepared using dilute nitric acid to clean the surfaces, followed by washing in reverse-osmosis purified water. Roughened SLA topography surfaces were prepared by blasting the titanium with corundum particles, followed by etching with HCl/H_2_SO_4_ as previously described [40].

### 4.2. Preparation of PRP and i-PRF

Whole blood samples were collected from the laboratory members involved in this study between the ages of 25 and 45. Blood was collected with the consent of participating members and a formal Institution Review Board (IRB) was not deemed necessary by our University. PRP was prepared according to Curasan protocol as previously described [46]. Briefly, 10 mL of whole blood with anticoagulant (ethylenediaminetetraacetic acid, EDTA) was centrifuged at 900× *g* for 5 min to separate PRP and platelet-poor plasma (PPP) portions from the red blood cell (RBC) fraction and then centrifuged a second time at 2000× *g* for 15 min to separate PRP from PPP. Finally, approximately 1 mL of PRP was isolated from 10 mL of whole blood. The i-PRF was produced as follows: 10 mL of whole blood without anticoagulant was immediately centrifuged at 700 rpm for 3 min with Choukroun PRF Duo Centrifuge (Process for PRF, Nice, France); 1 mL from the upper layer was designated the i-PRF. The collected PRP and i-PRF were then transferred to 6-well in vitro plastic culture dishes with 5 mL of culture media (Dulbecco’s Modified Eagle Medium (DMEM); HyClone, Thermo Fisher Scientific Inc., Beijing, China) and processed as further described.

### 4.3. Isolation of Human Gingival Fibroblasts

Gingival tissues were harvested from three healthy human donors undergoing third molar extraction, without periodontal disease as previously described [47]. Ethical approval and consent was obtained from all volunteers. All experiments were performed in triplicate with three independent experiments. Collected tissues were washed three times with phosphate buffered saline (PBS; 150 mM NaCl, 20 mM sodium phosphate, pH 7.2) containing 1% antibiotics (100 U/mL penicillin G, 100 µg/mL streptomycin, HyClone, Thermo Fisher Scientific Inc.) and cut into small pieces with sterilized surgical scissors. The gingival tissue pieces were then transferred into T25 tissue culture flasks containing minimal DMEM and were allowed to adhere for 2 h. Then, 3 mL of DMEM containing 20% fetal bovine serum (FBS; Gibco, Australia) and antibiotics was added. After one week when cells reached confluency, cells were trypsinized and cultured in DMEM with 10% FBS. Human gingival fibroblasts used for experiments were chosen from passages 3–7.

### 4.4. Cell Culture

Platelet concentrates including PRP and i-PRF were incubated for three days in a humidified 5% CO_2_ atmosphere at 37 °C and thereafter conditioned media was collected and utilized in future experiments as 20% of the total volume as previously described [13]. Human gingival fibroblasts were detached from tissue culture plastic using trypsin (HyClone) prior to reaching confluency. Cells were cultured in a humidified atmosphere at 37 °C in growth medium consisting of DMEM, 10% FBS and 1% antibiotics. Cells were seeded with 20% conditioned media from PRP and i-PRF, and contained within growth medium at a density of 10,000 cells for cell viability, cell migration, cell adhesion and proliferation experiments, and 50,000 cells for real-time PCR, collagen immunofluorescent staining per well in 24-well plates. For experiments lasting longer than five days, medium was replaced twice weekly. For experiments, 200 µL of PRP or i-PRF conditioned media was placed on implant surfaces for 5 min to allow a coating period followed by cell seeding.

### 4.5. Cell Viability

At 24 h post cell seeding, human gingival fibroblasts seeded on implant surfaces with/without PRP or i-PRF were evaluated using a live-dead staining assay. Live cells were stained with 2 µmol/L Calcein-AM (Dojindo, Japan) and dead cells were stained with 4 µmol/L propidium lodide (Sigma). The cells were washed with PBS and Live/Dead reagents were added and incubated for 15 min at 37 °C. Fluorescent images were quantified with an Olympus DP71 fluorescent microscope (Olympus Co., Tokyo, Japan). Thereafter, cells were expressed as percentages of live versus dead cells following cell culture growth with PRP and i-PRF.

### 4.6. Cell Migration Assay

The migration assay was performed using 24-well plates and polyethylene terephthalate cell culture inserts with a pore size of 8 µm (Costar, Corning Inc., Corning, NY, USA). The 20% platelet conditioned media in DMEM containing 10% FBS were filled into the lower compartment of the wells onto implant surfaces or controls. After starving the cells in DMEM containing 0.5% FBS for 12 h, 10,000 cells were resuspended and seeded in the upper compartment. After 24 h, cells were fixed with 4% formaldehyde for 15 min. Thereafter, cells were stained with 0.1% crystal violet solution (GoodBio Technology Co., Ltd., Wuhan, China) for 10 min. The upper side of the filter membrane was rinsed and gently wiped by a cotton swab to remove the cell debris. Images on the lower side of the filter were taken under an Olympus DP71 microscope (Olympus Co., Tokyo, Japan).

### 4.7. Cell Adhesion and Proliferation Assays

Gingival fibroblasts were seeded onto titanium discs in 24-well plates at a density of 10,000 cells per Ti structure and cultured for 2, 4 and 8 h for the adhesion assay. For counting the cell number, 4′,6-diamidino-2-phenylindole (DAPI) was applied to visualize the nuclei as previously described [48]. At each time point, the Ti structures were washed with PBS to remove non-attached cells and fixed in 4% formaldehyde for 10 min followed by staining with DAPI. Images were captured on an Olympus DP71 fluorescence microscope (Olympus Co., Tokyo, Japan). Ten fields of view were captured per sample and nuclei were counted using Image J software (Bethesda, MD, USA).

For the cell proliferation assays, HGFs were seeded on titanium discs in 24-well plates at a density of 10,000 cells per well with 20% culture medium from PRP, i-PRF. At time points 1, 3 and 5 days, cell number of HGFs was determined by the Cell Counting Kit-8 (Dojindo, Japan) and measured by a microplate reader scanning at 450 nm (PowerWave XS2, BioTek, Winooski, VT, USA) as previously described [49]. Both cell adhesion and proliferation experiments were performed in triplicate with three independent experiments performed.

### 4.8. Cell Morphology

HGFs were plated at a density of 10,000 cells on PT and SLA surfaces either with versus without PRP, i-PRF in a 24-well plate. At 8 h, cells were fixed using 4% formaldehyde followed by rinsing with PBS for 5 min. Then cells were stained with 5 µg/mL phalloidin-FITC (Fluorescein Isothiocyanate) (Sigma-Aldrich, St. Louis, MO, USA) for 1 h in dark conditions at 37 °C as previously described [40]. DAPI was used to visualize the cells’ nuclei. Images were captured from each surface on an Olympus DP71 fluorescence microscope (Olympus Co., Tokyo, Japan) for samples with and without PRP/i-PRF and compared for morphological differences.

### 4.9. Real-Time PCR Analysis

For real-time PCR experiments, 50,000 gingival fibroblasts were seeded onto PT and SLA surfaces with PRP or i-PRF in 24-well plates. After seven days of culture, total RNA was isolated from cells using AxyPrep™ Multisource Total RNA Miniprep Kit (AXYGEN, Union City, CA, USA) according to the manufacturer’s protocol. The RNA concentration was determined by a NanoDrop 2000 UV-Vis Spectrophotometer as previously described [50]. A total of 1 µg RNA solution was immediately reverse transcribed to cDNA using a First Strand cDNA Synthesis Kit (GeneCopoeia, Rockville, MD, USA) and the final volume is 100 µL. The sequences of primers for platelet-derived growth factor (*PDGF*), transforming growth factor-β (*TGF-β*), collagen type I α1 (*COL1a1*), fibronectin (*FN1*) and glyceraldehyde 3-phosphate dehydrogenase (*GAPDH*) genes of human are listed in Table 1. Real-time RT-PCR was performed using 20 µL final reaction volume of All-in-One™ qPCR Mix Kit (GeneCopoeia, Rockville, MD, USA) and the target gene expression was assayed on a CFX Connect™ Real-Time PCR Detection System. The ΔΔ*C*_t_ method was used to calculate gene expression levels relative to house-keeping gene GAPDH and normalized to control cells (blank well without Ti structure). Each sample contained pooled mRNA collected from three titanium surfaces and all samples were log-transformed. The experiments were performed in triplicate with three independent experiments.

### 4.10. Collagen Type I Staining

HGFs were plated at a density of 50,000 cells per structure in a 24-well plate. The culture medium was changed every three days. At seven days, Ti structures were washed with PBS and fixed with 4% formaldehyde for 10 min at room temperature (RT). Cells were then permeabilized with 0.5% Triton X-100 (Merck, Darmstadt, Germany) in PBS for 3 min at room temperature. Subsequently, cells were incubated with polyclonal rabbit to collagen type I (1:100, Boster Biological Technology Ltd., Wuhan, China) diluted in PBS containing 2% bovine serum albumin (BSA, Roche, Shanghai, China) for 1 h, followed by incubation with FITC-conjugated-goat-anti-rabbit (1:200, Invitrogen, Thermo Fisher Scientific, Waltham, MA, USA) diluted in PBS for 1 h. Finally, cells were stained with DAPI. After each step, the cells were washed with PBS three times. Images were taken from each surface on an Olympus DP71 fluorescence microscope (Olympus Co., Tokyo, Japan).

### 4.11. Statistical Analysis

Statistical analysis was analyzed by one-way analysis of variance (ANOVA) with Bonferroni test, using Graphpad Software v. 6 (Graphpad Software, La Jolla, CA, USA) and statistical significance was considered at *p* < 0.05. All data are expressed as the mean ± Standard Error (SE).

## Figures and Tables

**Table 1:** List of primer sequences for real-time PCR.

Gene	Primer Sequence (5′ to 3′)
*hPDGF F*	cacacctcctcgctgtagtattta
*hPDGF R*	gttatcggtgtaaatgtcatccaa
*hTGF-β F*	actactacgccaaggaggtcac
*hTGF-β R*	tgcttgaacttgtcatagatttcg
*hCOL1a1 F*	cccagccaagaactggtatagg
*hCOL1a1 R*	ggctgccagcattgatagtttc
*hFN1 F*	acctacggatgactcgtgctttga
*hFN1 R*	caaagcctaagcactggcacaaca
*hGAPDH F*	agccacatcgctcagacac
*hGAPDH R*	gcccaatacgaccaaatcc

## Abbreviations

i-PRF	Injectable Platelet-Rich Fibrin
PRP	Platelet-Rich Plasma
PT	Pickled Implant Surfaces
SLA	Sand-Blasted by Large Grit followed by Acid Etching Implant Surfaces
PDGF	Platelet-Derived Growth Factor
TGF-β	Transforming Growth Factor-β
VEGF	Vascular Endothelial Growth Factor
HCl	Hydrochloric Acid
H2SO4	Sulfuric Acid
EDTA	Ethylenediaminetetraacetic Acid
PPP	Platelet-Poor Plasma
RBC	Red Blood Cells
DMEM	Dulbecco’s Modified Eagle Medium
PBS	Phosphate Buffered Solution
FBS	Fetal Bovine Serum
DAPI	4′,6-Diamidino-2-phenylindole
HGF	Human Gingival Fibroblast
FITC	Fluorescein Isothiocyanate
COL1a1	Collagen type I α1
FN1	Fibronectin
GAPDH	Glyceraldehyde 3-Phosphate Dehydrogenase
RT	Room Temperature
