# Correction: Wang et al. Behavior of Gingival Fibroblasts on Titanium Implant Surfaces in Combination with Either Injectable-PRF or PRP. *Int. J. Mol. Sci.* 2017, *18*, 331

**DOI:** 10.3390/ijms27209019

**Published:** 2026-10-12

**Authors:** Xuzhu Wang, Yufeng Zhang, Joseph Choukroun, Shahram Ghanaati, Richard J. Miron

**Affiliations:** 1 The State Key Laboratory Breeding Base of Basic Science of Stomatology (Hubei-MOST) & Key Laboratory of Oral Biomedicine Ministry of Education, School & Hospital of Stomatology, Wuhan University, Wuhan 430079, China; wangxuzhu@whu.edu.cn; 2 Department of Oral Implantology, School and Hospital of Stomatology, Wuhan University, Wuhan 430079, China; 3 Pain Clinic, 06000 Nice, France; joseph@a-prf.com; 4 FORM, Frankfurt Oral Regenerative Medicine, Clinic for Maxillofacial and Plastic Surgery, Johann Wolfgang Goethe University, 60596 Frankfurt am Main, Germany; s.ghanaati@med.uni-frankfurt.de; 5 Department of Periodontology, College of Dental Medicine, Nova Southeastern University, Fort Lauderdale, FL 33328, USA; 6 Cell Therapy Institute, Collaborative Centre for Research, Nova Southeastern University, Fort Lauderdale, FL 33328, USA; 7 Department of Periodontics and Oral Surgery, University of Ann Arbor, Ann Arbor, MI 48109, USA

In the original publication [1], there was a mistake in Figures 2 and 6 as published. The Transwell picture of the PT + iPRF group in Figure 2A and the fluorescent image of the PT group in Figure 6A were misplaced. These errors arose from the inadvertent use of incorrect images during figure assembly. The images of the PT + iPRF group in Figure 2A and the PT group in Figure 6A have now been corrected. The corrected Figure 2 and Figure 6 appear below. The authors state that the scientific conclusions are unaffected. This correction was approved by the Academic Editor. The original publication has also been updated.

## Figures and Tables

**Figure 2:**
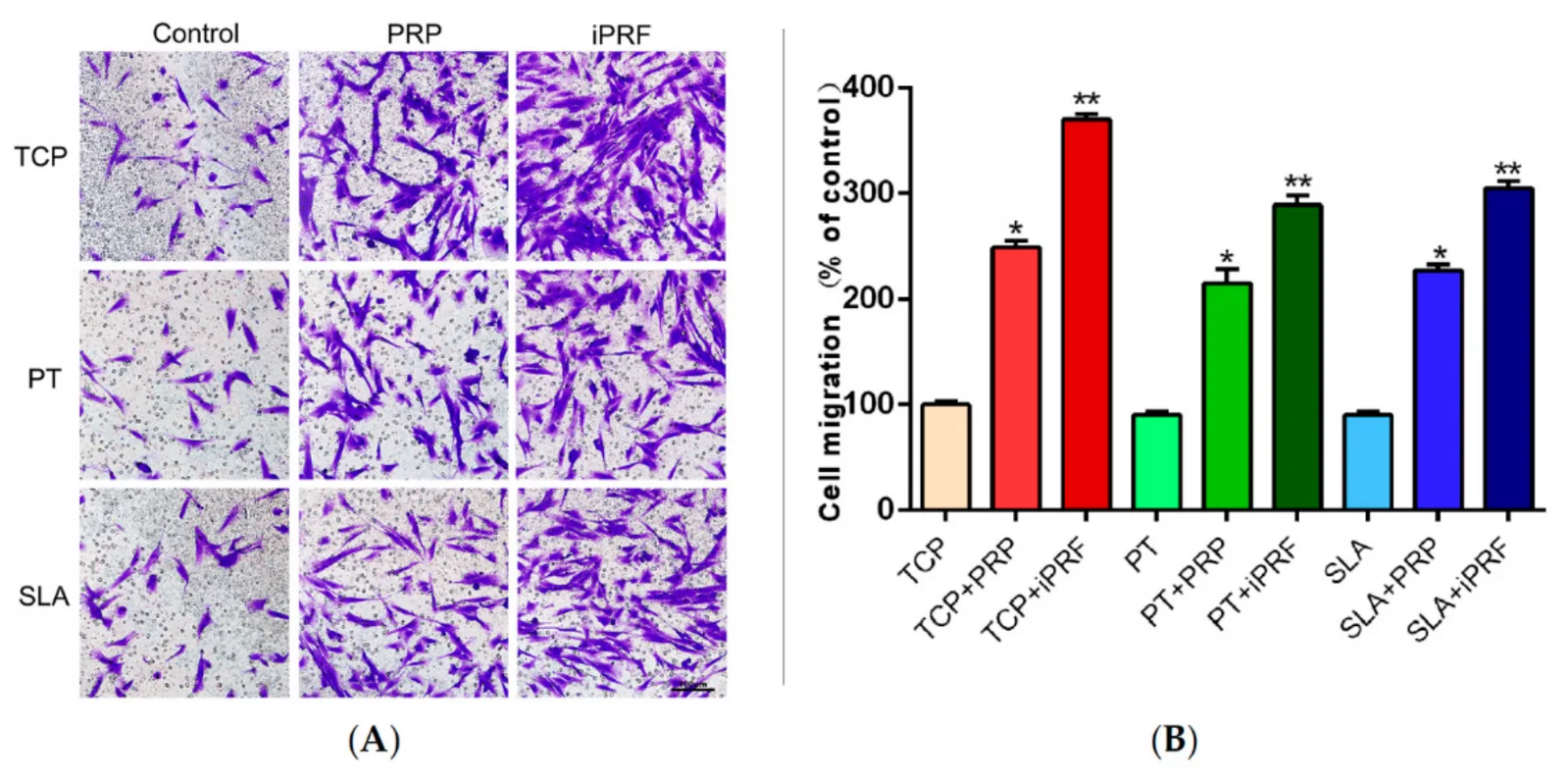
Effects of surface topography and PRP/i-PRF on the migration of human gingival fibroblasts. (**A**) Cell migration was assessed after 24 h. (Scale bars = 100 µm); (**B**) Cell migration was quantified by normalizing to the control TCP group. (* denotes significant difference between control and experimental group, *p* < 0.05; ** denotes significantly higher than all other groups, *p* < 0.05).

**Figure 6:**
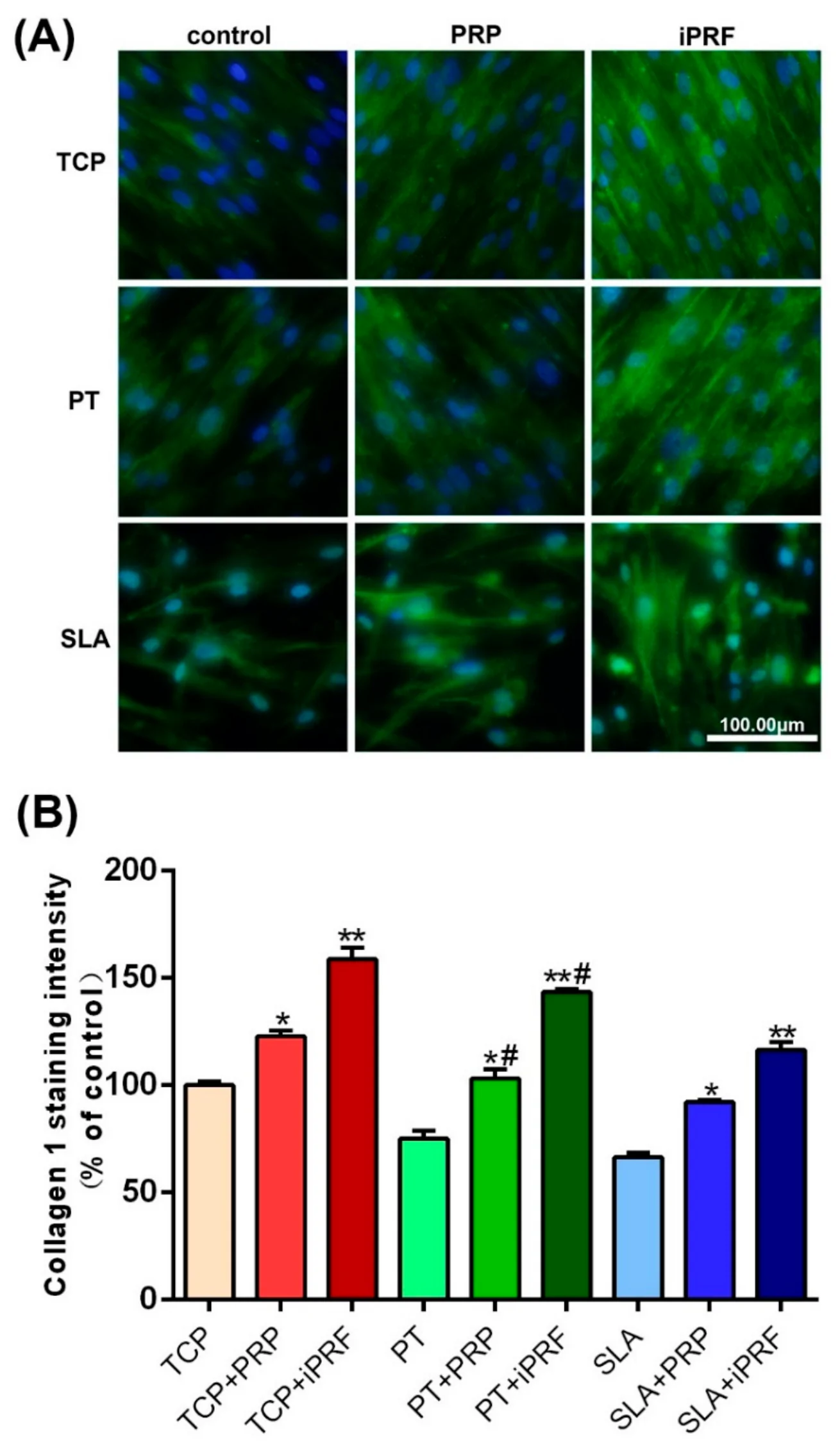
Immunofluorescent staining of collagen type 1 for human gingival fibroblasts seeded on TCP, PT and SLA surfaces with and without PRP and i-PRF at 7 days. (**A**) The merged fluorescent images of collagen type 1 staining (green) with DAPI staining (blue). (Scale bars = 100 µm); (**B**) Quantified values of collagen type 1 staining in comparison to control TCP sample (* denotes significant difference between control and experimental group, *p* < 0.05, ** denotes significantly higher than all other groups, *p* < 0.05, # denotes significant difference between PT and SLA surfaces, *p* < 0.05).

## References

[1] Wang X., Zhang Y., Choukroun J., Ghanaati S., Miron R.J. (2017). Behavior of Gingival Fibroblasts on Titanium Implant Surfaces in Combination with either Injectable-PRF or PRP. Int. J. Mol. Sci..

